# A comprehensive study integrating bioinformatics analysis and experimental results on HROB as a potential biomarker for the prognosis of lung adenocarcinoma

**DOI:** 10.1038/s41598-026-35798-7

**Published:** 2026-01-12

**Authors:** Fayan Zhang, Xiao Liu, Shengyu Zhou

**Affiliations:** 1 College of Medicine, Shandong University of Traditional Chinese Medicine, Jinan, China; 2 Department of Respiratory and Critical Care Medicine, Qilu Hospital, Cheeloo College of Medicine, Shandong University, Jinan, China; 3 Clinical Nursing Department, School of Nursing and Rehabilitation, Cheeloo College of Medicine, Shandong University, Jinan, China

**Keywords:** HROB, Biomarker, Lung adenocarcinoma, Immune infiltration, Cell cycle, Biomarkers, Diseases, Medical research, Molecular medicine, Oncology

## Abstract

HROB, which is a DNA-binding protein linked to various cancers, has an unclear role in lung adenocarcinoma (LUAD). To explore its clinical significance and potential pathogenesis, we analysed RNA-seq data from The Cancer Genome Atlas (TCGA) and the Gene Expression Omnibus (GEO) and focused on differential expression, survival impact, functional pathways, and immune infiltration. Our findings revealed that HROB mRNA expression is significantly elevated in LUAD tissues compared with normal lung tissues. High HROB expression is associated with more aggressive tumour characteristics and poorer prognosis of LUAD, with a hazard ratio of 1.815 being observed (*P* = 0.004), thus suggesting that HROB could serve as an independent prognostic biomarker. The results of the functional enrichment analyses indicated that HROB is involved in cell cycle regulation pathways. Notably, immune infiltration analysis revealed a significant correlation between HROB expression and Th2 cell infiltration (*R* = 0.626). We also constructed a protein-protein interaction (PPI) network using the STRING database and identified six candidate small-molecule agents that may target HROB. In vitro experiments demonstrated that the knockdown of HROB expression reduces LUAD cell proliferation and invasion while inducing changes in cell cycle progression. Overall, our study establishes HROB as a critical biomarker for LUAD prognosis and highlights its potential role as a therapeutic target.

**Supplementary Information:**

The online version contains supplementary material available at 10.1038/s41598-026-35798-7.

## Introduction

 Lung cancer has been a leading cause of cancer mortality in recent years. It is among the most abundant clinical cancer types, with 2 million new cases being diagnosed on a yearly basis^1^. Non-small cell lung cancer (NSCLC) is the predominant type and contributed to nearly 90% of all lung cancer cases in 2022^2^. Among these types, lung adenocarcinoma (LUAD) is the most common subtype, accounting for approximately 55–60% of NSCLC cases^3^. The substantial intertumoural genomic heterogeneity of LUAD^4^, coupled with the emergence of resistance to third-generation EGFR-TKIs^5^, constitutes a major obstacle to further improving therapeutic efficacy and demonstrates a novel clinical challenge, as well as exerting a considerable financial strain on health care systems throughout the world^6^. These findings underscore the urgent need for identifying new biomarkers and therapeutic targets that could improve patient outcomes and enhance clinical management^7^.

The rapid evolution of omics technologies has markedly accelerated the elucidation of oncogenic mechanisms and the discovery of tumour biomarkers. Integrated multiomics analyses, such as genomics, transcriptomics, and proteomics, offer an unprecedented capacity to elucidate tumour heterogeneity, refine prognosis, and tailor individualised therapies^8,9^. The systematic integration of omics datasets further elucidates intricate molecular networks, thereby enabling efficient identification of diagnostic, therapeutic, and prognostic biomarkers that reinforce precision oncology^10^. In the present study, by the utilisation of publicly accessible databases (including the TCGA and GEO databases) combined with comprehensive bioinformatics analyses and experimental validation, we conducted a systematic investigation into the functional role of HROB in lung adenocarcinoma. HROB is a homologous recombination factor with an OB-fold (also known as C17orf53) located on chromosome 17. This DNA-binding protein is known to recruit the MCM8/MCM9 helicase to sites of DNA damage^11^. HROB is crucial for repairing DNA damage and maintaining genomic integrity^12^; moreover, it has been linked to several types of cancer, including hepatocellular carcinoma^13^, breast cancer^14^, and clear cell renal cell carcinoma^15^. Despite these findings, the exact mechanisms by which HROB influences the progression of LUAD and patient outcomes are not yet fully understood.

We utilised datasets from The Cancer Genome Atlas (TCGA) and the Gene Expression Omnibus (GEO) to assess HROB expression levels in LUAD and their relationships with clinical outcomes. This integrative approach allowed us to clarify the potential role of HROB as an independent prognostic factor in LUAD. Additionally, we explored the functional implications of HROB expression for cellular processes, including its connections to small-molecule drugs and supporting experimental verification data. In conclusion, our investigation aims to provide a detailed understanding of the function of HROB in lung adenocarcinoma. This study also offers valuable insights that could enhance prognostic evaluations and treatment strategies for patients with LUAD.

## Materials and methods

### Collection of datasets

In this investigation, RNA sequencing (RNA-seq) data for 33 cancer types, along with corresponding normal tissue samples, were obtained from the TCGA database (https://portal.gdc.cancer.gov/). Additionally, clinicopathological data for LUAD patients were extracted from the same TCGA database, with the data including 598 tumour samples and 59 adjacent noncancer samples. Samples with missing clinical records or incomplete expression data were excluded to ensure high-quality and consistent analytical datasets. RNA quality thresholds were rigorously applied during cohort curation. For the TCGA datasets, samples with an RNA integrity number (RIN) < 7.0 were excluded based on standard protocols^16^. The raw data underwent normalisation by using transcripts per million (TPM), followed by further analysis using log2 (TPM + 1). Furthermore, expression profiling microarray datasets (specifically GSE10072 and GSE41271) were retrieved from the GEO database (http://www.ncbi.nlm.nih.gov/projects/geo/). The GSE10072 dataset includes 58 tumour samples and 49 normal samples. The GSE41271 dataset includes 183 samples from lung adenocarcinoma patients with complete survival records, including overall survival (OS) status and follow-up duration. GSE10072 was profiled on the Affymetrix Human Genome U133 Plus 2.0 array and retained only samples with an RIN ≥ 7, whereas GSE41271 was generated on the Illumina HumanWG-6 v3.0 expression BeadChip with a threshold RIN value of ≥ 4.0. Statistical analyses of these datasets were performed by using the Xiantao database (https://www.xiantaozi.com/), which is an online bioinformatics platform based on R software.

### Analyses of HROB expression and survival

The analysis of differentially expressed HROB genes (DEGs) between tumour tissues and adjacent normal tissues from different tumours in the TCGA and GSE10072 datasets, as well as across various clinicopathological characteristic groups within LUAD patients, was conducted and visualised by using the Xiantao tool, wherein the “stats”, “car”, and “ggplot2” packages were utilised. Patients with LUAD were divided into two groups based on the median expression levels of HROB mRNA: a low-expression group (0–50%) and a high-expression group (50–100%). To assess survival outcomes, we used both Kaplan-Meier and Cox regression methods. We compared overall survival (OS), disease-specific survival (DSS), and progression-free interval (PFI) between the two HROB expression groups by using the Xiantao tool (specifically the “Survival” package). Furthermore, we compared OS among different smoker subgroups of LUAD patients and within the GSE41271 dataset. Kaplan-Meier survival plots were created by using the Xiantao tool, with the “Survminer” and “ggplot2” packages being utilised.

### Receiver operating characteristic curve

We employed receiver operating characteristic (ROC) curves to assess the diagnostic efficacy of HROB in LUAD. The ROC analysis was conducted and visualised via the Xiantao tool, with the “pROC”, “timeROC”, and “ggplot2” packages being utilised.

### Univariate and multivariate Cox regression analyses

The proportional hazards hypothesis was evaluated by using the Xiantao tool from the “survival” package, followed by both univariate and multivariate Cox regression analyses to assess the independent prognostic significance of elevated HROB expression (in addition to various clinicopathological characteristics) on the OS of LUAD patients. Factors demonstrating p values of less than 0.05 in the univariate Cox regression analysis were included in the multivariate Cox regression analysis to further clarify their independent prognostic importance. Additionally, forest plots and survival point charts were generated by using the Xiantao tool with the “ggplot2” package.

### Generation and prediction of prognostic models

The independent prognostic factors identified from the multivariate Cox regression analysis were utilised to construct a nomogram model with the Xiantao tool from the “rms” package, with an aim of predicting the 1-, 3-, and 5-year OS for LUAD patients. Calibration analysis, time-dependent AUC metrics, and corresponding visualisations were conducted by using the Xiantao tool with the “rms” and “timeROC” packages, thereby highlighting the differences between the predicted and actual probabilities across various time points.

### Gene Ontology, KEGG, and GSE analyses

The DEGs were statistically evaluated by employing the “DEseq2” and “limma” packages. Volcano plots illustrating these findings were generated by using the Xiantao tool with the “ggplot2” package. To further explore the differences in biological functions and signalling pathways between the high and low HROB expression groups, the DEGs were subjected to Gene Ontology (GO) and Kyoto Encyclopedia of Genes and Genomes (KEGG) analyses, wherein the Xiantao tool was again utilised (particularly the “clusterProfiler” package). Genes demonstrating absolute log2-fold changes (FC) greater than 1 and with adjusted p values of less than 0.05 were classified as DEGs and subsequently used for GO and KEGG analyses^17^. For gene set enrichment analysis (GSEA), the gene set “c2.cp.all.v2022.1.Hs.symbols.gmt [All Canonical Pathways] (3050)” from the Molecular Signatures Database (MSigDB) was chosen as the reference set, with items exhibiting a false discovery rate (FDR) of less than 0.25 and an adjusted p value of less than 0.05 considered to be significantly enriched. Multiple testing correction was algorithmically implemented through the built-in Benjamini-Hochberg FDR procedure of the GSEA software. The results from the GO, KEGG, and GSEA enrichment analyses were visualised by using the Xiantao tool and the “ggplot2” package.

### Immune infiltration analysis

We conducted immune infiltration analysis by using the “Estimate” package in R software (version 4.2.1) to assess stromal scores, immune scores, and estimate scores within the LUAD microenvironment, thereby differentiating between high and low levels of HROB expression. Additionally, correlation analysis between tumour purity and HROB expression was performed. TCGA-LUAD gene expression profiles were obtained from the UCSC Xena browser (https://tcga.xenahubs.net/download/TCGA.LUAD.sampleMap/HiSeqV2.gz). Tumour purity was calculated by using the ESTIMATE algorithm (v1.0.13) implemented in the Xiantao bioinformatics toolkit. The association between HROB expression and tumour purity was evaluated through correlation scatterplots generated via the “Correlation Scatter Plot” module in the Xiantao tool. To explore the immune infiltration of 24 different immune cell types in LUAD, we performed single-sample gene set enrichment analysis (ssGSEA), which allowed us to calculate enrichment scores for each immune cell in tumour samples based on their associated marker genes, whereby we utilised the Xiantao tool with the “GSVA” package. We applied the Wilcoxon rank-sum test to compare the immune cell enrichment scores between the high and low HROB expression groups. Additionally, we performed correlation analyses between HROB expression and various immune cells by using Spearman correlation analysis. The results were visualised with the Xiantao tool (specifically via the “ggplot2” package) for effective graphical representation.

### Screening of HROB-coexpressed genes in LUAD

We employed the Spearman’s correlation test to assess the relationships between individual genes and HROB in LUAD. We identified genes exhibiting adjusted p values of less than 0.05 and ranked them based on their correlation coefficients. From this analysis, we selected the top 50 genes that demonstrated either positive or negative correlations with HROB for further investigation. To visualise these relationships, we created a coexpression heatmap highlighting the top eight genes that were most strongly correlated with HROB, with the Xiantao tool and the “ggplot2” package being utilised for graphical representation.

### Construction of the protein-protein interaction network and identification of hub genes

To investigate the interactions among coexpressed genes related to HROB, we utilised the STRING database (https://stringdb.org/) to construct a protein-protein interaction (PPI) network, with the default parameters provided by the platform being used. Following the construction of the network, we employed Cytoscape software for visualisation purposes. Moreover, we identified the top ten hub genes using the CytoHubba plugin.

### Prediction of therapeutic Small-Molecule agents

Potential candidate small molecules for the treatment of LUAD were identified by using the Connectivity Map (cMAP) database from the Broad Institute (https://portals.broadinstitute.org/cmap). We uploaded all of the commonly shared DEGs from the TCGA database into the cMAP database. Molecules that exhibited high negative values were deemed to have potential therapeutic effects against LUAD. For further analysis, detailed information and structural representations of these compounds were sourced from PubChem (https://pubchem.ncbi.nlm.nih.gov) and ChemDraw.

### Cell culture, rt-qPCR, and cell transfection

The A549 cell line (which is a well-known lung cancer cell line) and the 16HBE cell line (which represents normal lung cells) were sourced from the Research Center of Shandong Cancer Hospital and Institute in China. Both cell lines were cultured in RPMI-1640 medium (Gibco, USA) supplemented with 10% foetal bovine serum and maintained in a CO_2_ incubator at 37 °C. For the transfection of small interfering RNAs (siRNAs), A549 cells were first seeded in 6-well plates and transfected with Lipofectamine 3000 (Invitrogen, USA) 24 h after seeding, following the manufacturer’s instructions. Quantitative PCR (qPCR) was performed by using a FastQuant cDNA First Chain Synthesis Kit (de-genome) (KR116) to compare the expression levels of HROB between 16HBE cells and A549 cells. The expression levels were analysed by using the 2 − ΔΔCt method, with β-actin serving as the reference control. The designed primer sequences for HROB were as follows: forward, 5′-ACAGGTTGCTGCTGGAGAC-3′; reverse, 5′-GGCTGAGATGGCTTGAGGAA-3′. The sequences of the utilised actin primers were as follows: forward, 5′-ACACTGTGCCCATCTACG-3′; reverse, 5′-TGTCACGCACGATTTCC-3′. In a separate siRNA transfection process, A549 cells were again plated in 6-well plates and transfected with Lipofectamine 2000 (11668-019) (Invitrogen, USA) 24 h after seeding, following the manufacturer’s guidelines. The specific target sequences for the siRNAs included shHROB-1 (GAGGGTTCAGTATTGGCTAAA), shHROB-2 (CCAACAACCTGGTCCATATTT), and shHROB-3 (GATGCAGAGAACCGGTTTACT). A549 cells were transfected by using the Lipofectamine 2000 Transfection Reagent (Invitrogen, USA) according to the manufacturer’s protocol.

### Cell proliferation assay

A cell proliferation assay was performed by using CCK-8 solution (CCK-8 Kit; MeilunBio, Dalian, China) according to the manufacturer’s guidelines. Briefly, A549 cells with stable sh-HROB expression and sh-NC cells were plated in 96-well plates at a density of 1 × 10³ cells per well and incubated for 24, 48, or 72 h. After the incubation period, the cells were treated with CCK-8 solution and allowed to incubate for an additional 72 h. The absorbance was then measured at 450 nm by using a microplate reader.

### Transwell assay

A549 cells were seeded at a density of 5 × 10⁵ cells per well in 6-well plates and incubated at 37 °C with 5% CO_2_ until they reached approximately 90% confluence over a 24-hour period. In this setup, 5 × 10⁵ cells were placed in the upper chamber and suspended in serum-free medium, whereas the lower chamber was filled with medium containing 10% foetal bovine serum (FBS). After the cells were allowed to incubate for 24 h, those cells that had migrated to the lower chamber were fixed by using 4% paraformaldehyde for 15 min. Following fixation, the cells were stained with crystal violet (Solarbio, Beijing, China) for 5 min. Finally, the migrated cells were imaged and counted to determine the invasive ability of the A549 cells.

### Cell cycle assay

Cell cycle analysis was performed by using a commercial staining kit from Multisciences Biotechnology (Hangzhou, China). This analysis compared lung cancer A549 cells that were transfected with sh-HROB to those that were transfected with sh-NC. First, 1 × 10⁴ cells per well were seeded into 96-well plates and cultured for 24 h. The cells were then fixed overnight in 70% ethanol at 4 °C. After two washes with PBS, the cells were stained with propidium iodide (PI) at a concentration of 0.05 mg/mL for 15 min in the dark at 4 °C. Flow cytometric analysis was conducted by using a CytoFLEX instrument from Beckman (California, USA), and the resulting data were processed by using Cell Quest software.

### Statistical analysis

Statistical analysis was conducted via the Wilcoxon signed-rank test and logistic regression to investigate the relationships between the clinical features of LUAD and HROB expression. To assess the associations between HROB expression and the baseline clinicopathological characteristics of the patients, the chi-square test was performed with the Xiantao tool (specifically via the “stats” package). For the statistical analyses of the in vitro experiments, GraphPad Prism 7.0 was used, with comparisons between two groups being performed by using the Student’s t test, and the results are presented as the mean ± standard deviation (SD). A p value of less than 0.05 was deemed to indicate statistical significance, with significance thresholds defined as follows: * *P* < 0.05; ** *P* < 0.01; and *** *P* < 0.001.

## Results

### Elevated HROB expression in LUAD and its correlation with prognosis

**Fig. 1:**
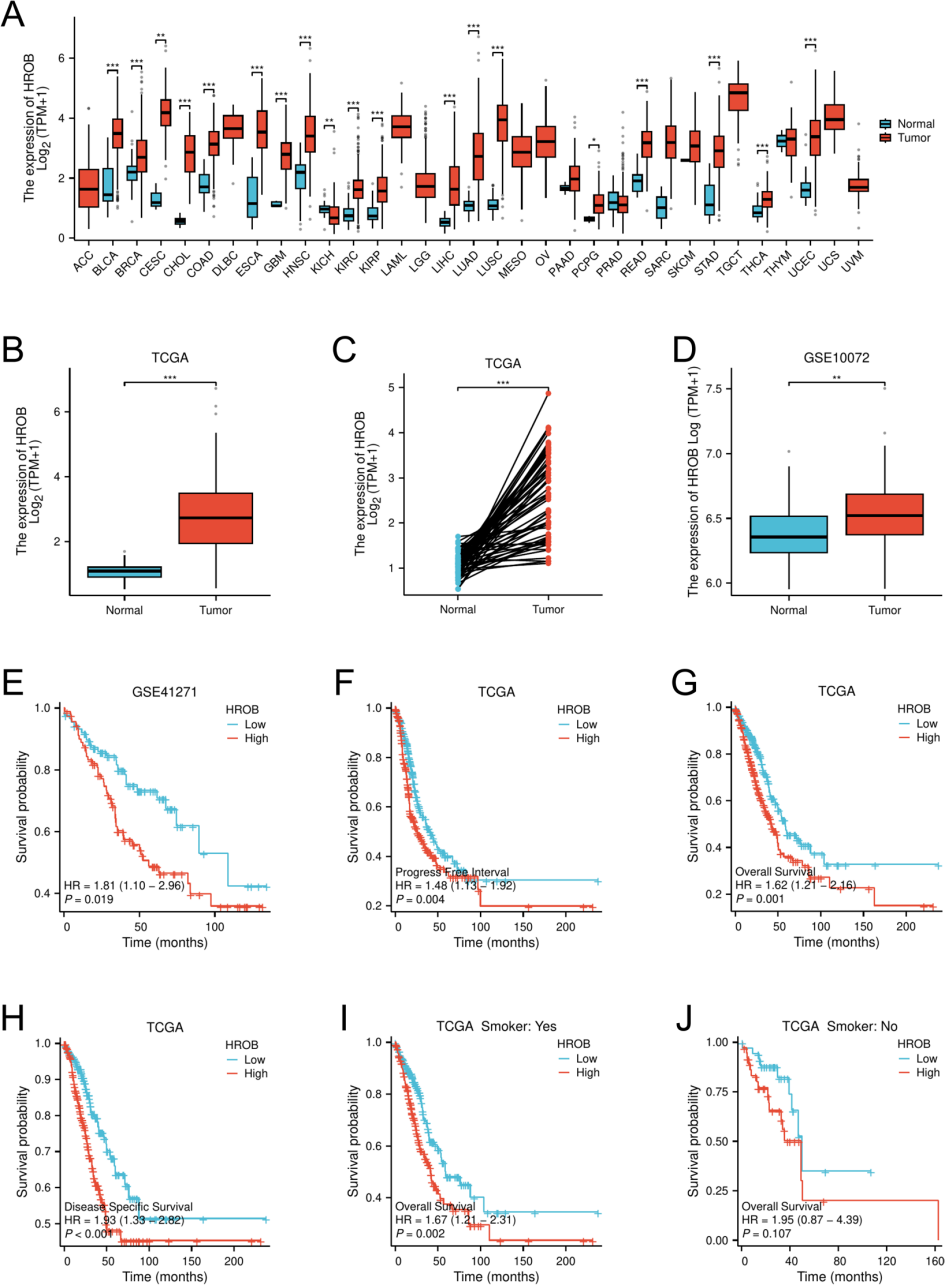
The variation in HROB expression levels across different tumour types and their significant correlations with the prognosis of lung adenocarcinoma (LUAD) are illustrated. (**A**) The TCGA database reveals HROB expression categorised as either high or low across various tumour types. (**B**) A comparison of HROB expression between LUAD cancer tissues and their corresponding normal tissues from the TCGA database is presented. (**C**) The HROB expression level is observed to be elevated in LUAD tumours compared to adjacent noncancerous tissues from the TCGA database. (**D**) HROB expression is greater in LUAD tumours than in normal tissues in the GSE10072 dataset. (**E**) Overall survival (OS) probabilities for LUAD patients are depicted in the GSE41271 dataset. (**F**) Progression-free interval (PFI) probabilities for LUAD patients are indicated based on the TCGA database. (**G**) The OS probabilities for LUAD patients from the TCGA database are shown. (**H**) The disease-specific survival (DSS) probabilities for LUAD patients in the TCGA database are presented. (**I**) OS probabilities for LUAD patients who are smokers are illustrated from the TCGA database. (**J**) OS probabilities for LUAD patients who are nonsmokers are also detailed in the TCGA database. Statistical significance is indicated as follows: ∗*P* < 0.05; ∗∗*P* < 0.01; and ∗∗∗*P* < 0.001 compared with the control group.

HROB expression was significantly elevated across various tumour types, including bladder cancer (BLCA), breast cancer (BRCA), and lung squamous cell carcinoma (LUSC). In contrast, we observed a notable decrease in HROB expression in kidney chromophobe (KICH) compared with normal tissues, as indicated by the TCGA data (Fig. 1A). Different tumour type-specific log2-fold changes (log2FC) for HROB are shown in the Supplementary Table (Supplementary Information: Table S1). Specifically, in LUAD, HROB expression was significantly greater in both unpaired (*P* < 0.001) and paired (*P* < 0.001) comparisons of tumour tissues compared to normal tissues (Fig. 1B and C). To further validate our findings, we analysed independent external datasets from the GEO and confirmed that HROB expression was significantly lower in normal tissues compared to LUAD tissues in the GSE10072 dataset (*P* < 0.001) (Fig. 1D). Kaplan-Meier survival analysis demonstrated that LUAD patients with high HROB expression experienced poorer OS, DSS, and PFI. Additionally, analysis of the GSE41271 dataset supported the correlation between elevated HROB expression and poor OS (Fig. 1E). Notably, subgroup analysis revealed that smokers with LUAD demonstrated worse OS, whereas nonsmokers did not exhibit the same trend (Fig. 1I and J). Our comprehensive analysis across multiple datasets consistently indicates that increased HROB expression is predictive of adverse clinical outcomes in patients with LUAD.

### Association of high HROB expression with malignant phenotypes in LUAD

We explored the associations between HROB expression and various clinical characteristics and pathological outcomes in patients with LUAD, which are summarised in Table 1. High levels of HROB expression were significantly linked to several clinical factors, including clinical T stage (*P* = 0.005), M stage (*P* = 0.030), pathological stage (*P* = 0.008), primary therapy outcome (*P* = 0.035), number of pack years smoked *(P* < 0.001), and age (*P* = 0.036). However, we did not observe a significant correlation between HROB expression and N stage, sex, or smoking status. Additionally, logistic regression analysis reinforced the significant relationships between HROB and age (*P* = 0.035), pathological TNM stage (*P* < 0.001, *P* = 0.025, and *P* = 0.036, respectively), primary therapy outcome (*P* = 0.015), and pathological stage (*P* = 0.043), as shown in Table 2. These results indicate that HROB may serve as an independent factor associated with LUAD.

**Table 1:** Demographic and clinical data of LUAD patients.

Rank	Score	Name	Description
1	−1.7648	PP-2	ATP-competitive inhibitor of the Src family of protein tyrosine kinases.
2	−1.7595	preladenant	Stimulates a T-cell-mediated immune response against tumour cells.
3	−1.6672	tivantinib	Small molecule inhibitor of c-Met with potential antineoplastic activity.
4	−1.6125	lenvatinib	Receptor tyrosine kinase (RTK) inhibitor.
5	−1.5875	filgotinib	Inhibitor of the tyrosine kinase Janus kinase 1 (JAK1).
6	−1.5791	orteronel	Being investigated for the treatment of prostate cancer.

**Table 2:** Logistic regression analysis on the relationship between HROB expression and clinical characteristics.

Characteristics	Low expression of HROB	High expression of HROB	*P* value
n	269	270	
Pathological T stage, n (%)			0.005
T1	107 (20%)	69 (12.9%)	
T2	129 (24.1%)	163 (30.4%)	
T3	22 (4.1%)	27 (5%)	
T4	10 (1.9%)	9 (1.7%)	
Pathological N stage, n (%)			0.069
N0	184 (35.2%)	166 (31.7%)	
N1	42 (8%)	55 (10.5%)	
N2	30 (5.7%)	44 (8.4%)	
N3	0 (0%)	2 (0.4%)	
Pathological M stage, n (%)			0.030
M0	184 (47.2%)	181 (46.4%)	
M1	7 (1.8%)	18 (4.6%)	
Pathological stage, n (%)			0.008
Stage I	165 (31.1%)	131 (24.7%)	
Stage II	53 (10%)	72 (13.6%)	
Stage III	37 (7%)	47 (8.9%)	
Stage IV	8 (1.5%)	18 (3.4%)	
Primary therapy outcome, n (%)			0.035
PD	24 (5.3%)	47 (10.5%)	
SD	19 (4.2%)	19 (4.2%)	
PR	3 (0.7%)	3 (0.7%)	
CR	177 (39.4%)	157 (35%)	
Smoker, n (%)			0.084
No	45 (8.6%)	32 (6.1%)	
Yes	214 (40.8%)	234 (44.6%)	
Number pack years smoked, n (%)			< 0.001
< 40	105 (28.5%)	83 (22.5%)	
≥ 40	68 (18.4%)	113 (30.6%)	
Gender, n (%)			0.180
Female	152 (28.2%)	137 (25.4%)	
Male	117 (21.7%)	133 (24.7%)	
Age, n (%)			0.036
≤ 65	118 (22.7%)	139 (26.7%)	
> 65	145 (27.9%)	118 (22.7%)	

**Fig. 2:**
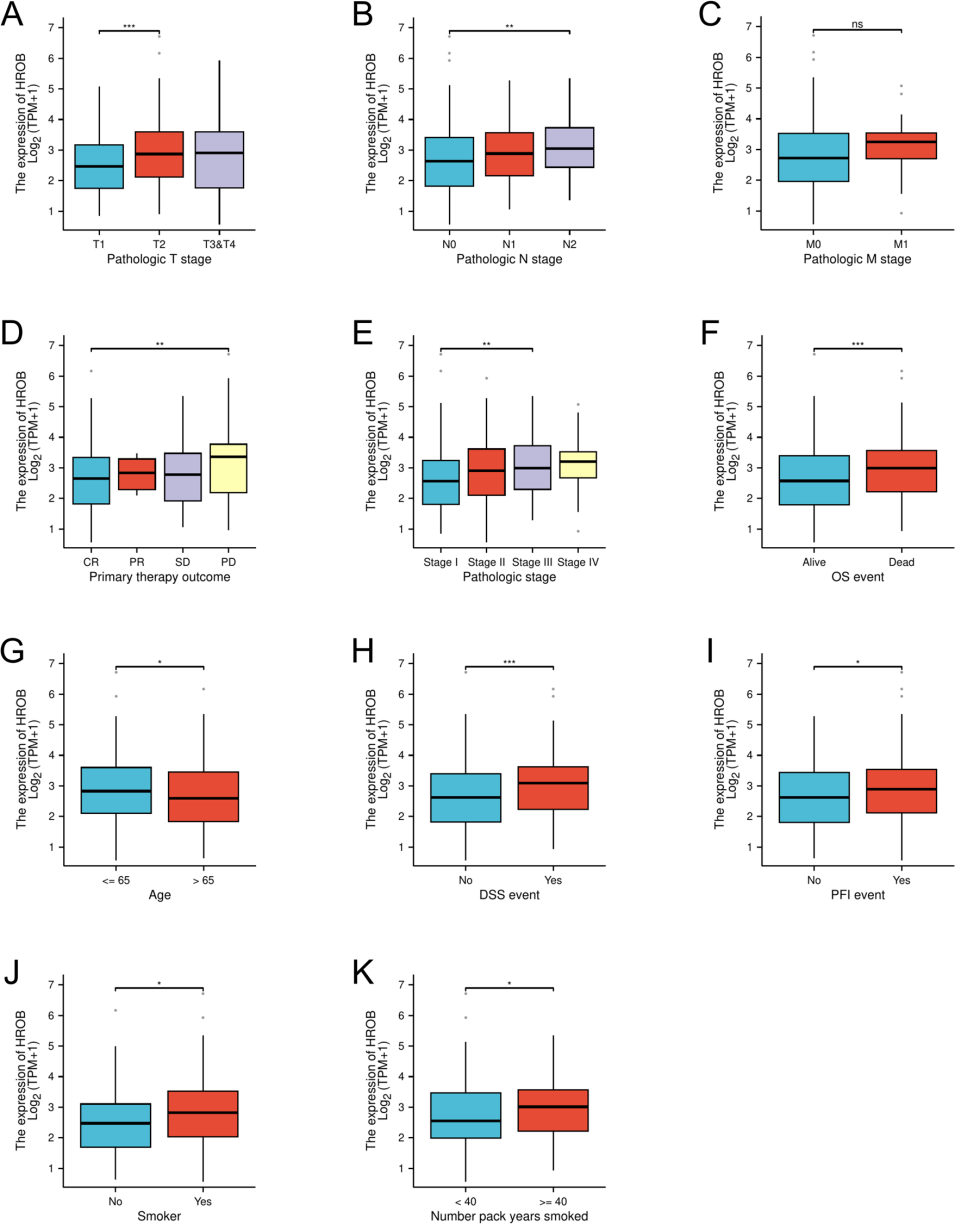
Significant correlations were demonstrated between HROB expression and various clinical characteristics of LUAD. (**A**) T stages, (**B**) N stage, (**C**) M stage, (**D**) primary therapy outcomes, (**E**) pathological stage, (**F**) OS events, (**G**) age, (**H**) DSS events, (**I**) PFI events, (**J**) smoking status, and (**K**) number pack years smoked were analysed. Statistical significance is denoted as follows: ∗*P* < 0.05; ∗∗*P* < 0.01; and ∗∗∗*P* < 0.001 compared with the low-HROB expression group.

Our results also revealed that HROB expression was significantly lower in the T1 stage compared to the T2 stage (*P* < 0.001), whereas no significant differences were observed between the T1 stage and the T3 or T4 stage (Fig. 2A). Similarly, HROB expression levels were significantly higher in the N2 stage than in the N0 stage (*P* < 0.01) (Fig. 2B). However, we observed no significant difference in HROB expression between the M0 and M1 stages (*P* > 0.05) (Fig. 2C), which may be due to the limited number of metastatic cases included in our analysis. Additionally, HROB expression was notably higher in patients with progressive disease (PD) compared to those who achieved a complete response (CR) (*P* < 0.01), with no significant differences being observed between CR and partial response (PR) or stable disease (SD) (*P* > 0.05) (Fig. 2D). Furthermore, compared with that in stage I patients, HROB expression in stage III patients was significantly elevated (*P* < 0.01) (Fig. 2E). When HROB expression was compared across other clinicopathological characteristics, we observed increased levels in subgroups of patients aged 65 years or younger (Fig. 2G), as well as in those who experienced OS events (Fig. 2F), DSS events (Fig. 2H), and PFI events (Fig. 2I). Notably, HROB expression was significantly higher in smokers than in nonsmokers (*P* < 0.01) (Fig. 2J); moreover, compared with patients with fewer than 40 pack years of smoking, patients with a smoking history of more than 40 pack years also exhibited elevated HROB levels (Fig. 2K). These findings highlight a clear association between increased HROB expression and various malignant phenotypes in LUAD.

### Predictive accuracy of HROB expression in LUAD

**Fig. 3:**
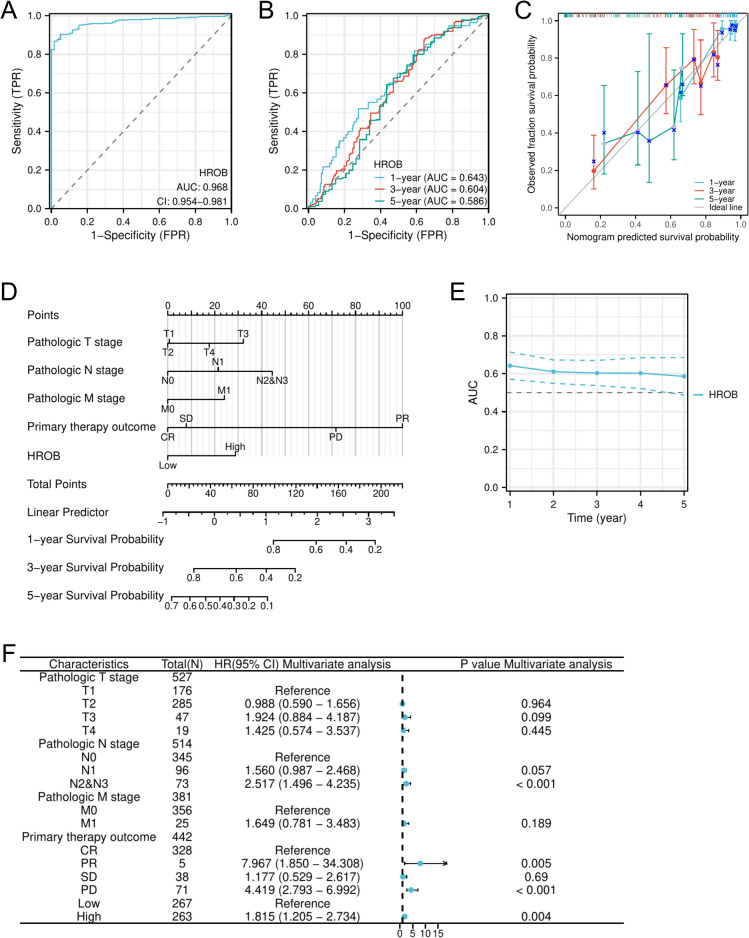
The prognostic significance of HROB expression levels in LUAD was assessed. (**A**) The receiver operating characteristic (ROC) curve for HROB in LUAD is illustrated. (**B**) Time-dependent ROC curves for HROB expression in LUAD are shown. (**C**) Calibration curves that demonstrate the alignment between the predicted and actual survival probabilities in the nomogram are presented. (**D**) A nomogram that integrates HROB and additional prognostic factors in LUAD derived from the TCGA database is depicted. (**E**) The time-dependent area under the curve (AUC) for HROB expression in LUAD is presented. (**F**) A forest plot illustrating OS via multivariate Cox regression analysis of LUAD data from the TCGA database is included. Statistical significance is indicated as follows: ∗*P* < 0.05; ∗∗*P* < 0.01; and ∗∗∗*P* < 0.001 compared with the control group.

The predictive potential of HROB expression in LUAD is illustrated in Fig. 3. ROC curve analysis revealed an AUC value of 0.968 (95% CI: 0.954–0.981), thus suggesting that HROB expression is an excellent prognostic marker for LUAD, with values near 1 indicating high predictive accuracy (Fig. 3A). Additionally, the time-dependent ROC curve analysis and time-dependent AUC confirmed the ability of HROB to reliably predict overall survival, with AUC values of 0.643 at one year, 0.604 at three years, and 0.586 at five years being observed (Fig. 3B and E). Univariate Cox regression analysis revealed a correlation between increased HROB expression and reduced overall survival (OS) (HR: 1.616; 95% CI: 1.208–2.163; *P* = 0.001). Moreover, multivariate Cox regression analysis (which considered other clinicopathological variables) revealed HROB as an independent prognostic risk factor for LUAD patients (HR: 1.815; 95% CI: 1.205–2.734; *P* = 0.004) (Table 3). Other clinicopathological factors, including N2 and N3 (HR: 2.517; 95% CI: 1.496–4.235; *P* < 0.001), PR (HR: 7.967; 95% CI: 1.850–34.308; *P* = 0.005), and PD (HR: 4.419; 95% CI: 2.793–6.992; *P* < 0.001), were also recognised as independent prognostic indicators (Fig. 3F). The nomogram demonstrated that the prognostic predictions based on HROB expression levels were superior to those derived from the M stage (Fig. 3D). The specific formula for scoring each variable was as follows:

**Table 3:** Univariate and multivariate Cox regression analyses of clinical characteristics associated with overall survival.

Characteristics	Total (*N*)	OR (95% CI)	*P* value
Gender (female vs. male)	539	0.793 (0.565–1.113)	0.180
Age (> 65 vs. ≤ 65)	520	0.691 (0.489–0.976)	**0.036**
Pathological T stage (T2 & T3 vs. T1)	517	1.951 (1.347–2.826)	**< 0.001**
Pathological N stage (N1 & N2 vs. N0)	521	1.524 (1.054–2.204)	**0.025**
Pathological M stage (M1 vs. M0)	390	2.614 (1.066–6.409)	**0.036**
Primary therapy outcome (PD & SD vs. CR & PR)	449	1.727 (1.113–2.679)	**0.015**
Pathological stage (Stage III & Stage IV vs. Stage I & Stage II)	531	1.551 (1.014–2.374)	**0.043**

$$\:\mathrm{r}\mathrm{i}\mathrm{s}\mathrm{k}Score\:=\:\sum\:_{i}Coefficient\:\left({gene}_{i}\right)\mathrm{*}mRNA\:Expression\:\left({gene}_{i}\right)$$

The calibration curves for the one-year, three-year, and five-year survival rates demonstrated strong agreement between the predicted probabilities and the actual observed outcomes, as illustrated in Fig. 3C.

### Functional enrichment analysis of DEGs between the high- and low-HROB-expression groups in LUAD

**Fig. 4:**
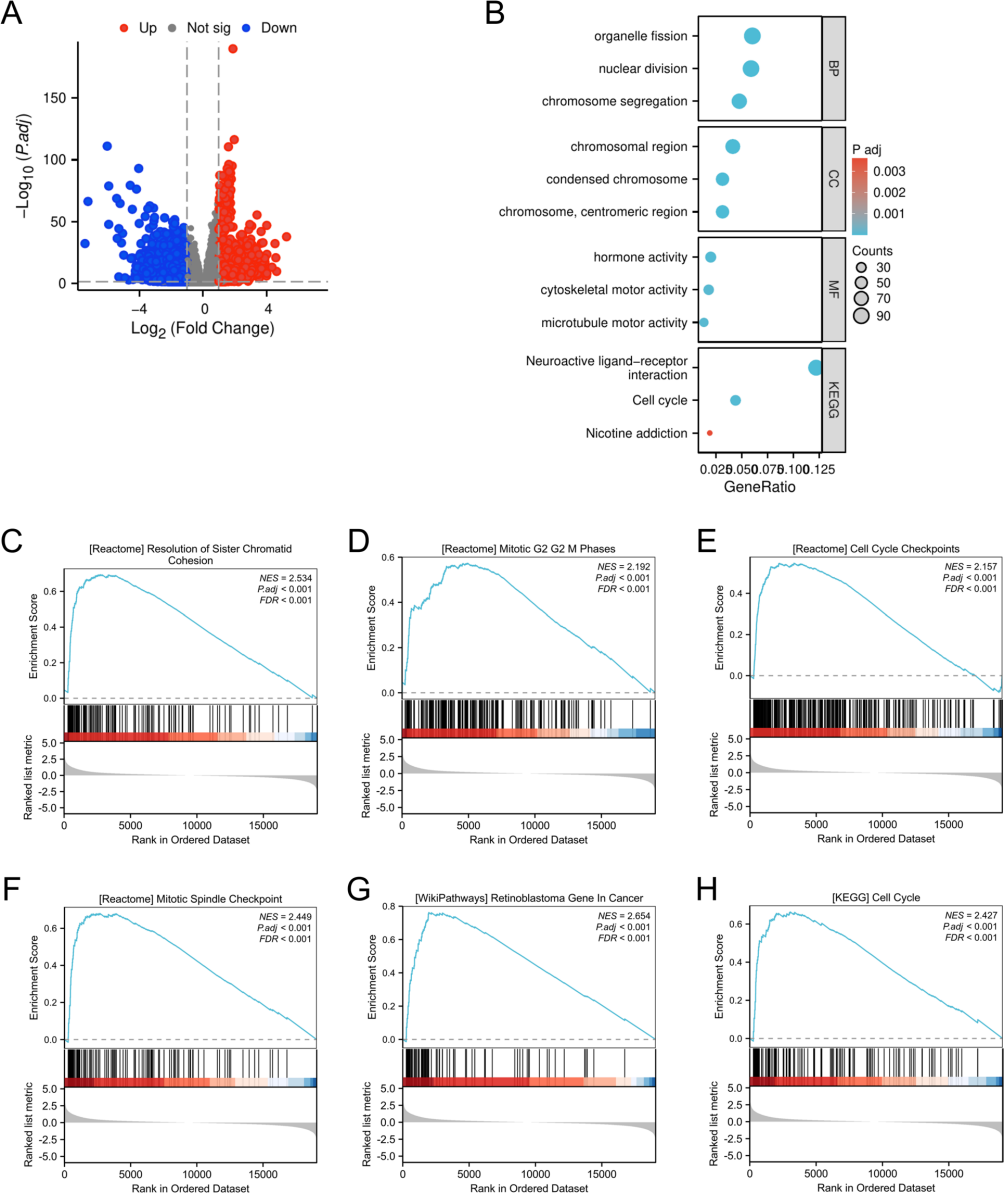
A functional enrichment analysis of differentially expressed genes (DEGs) between the high- and low-HROB expression groups was performed. (**A**) A volcano plot of the DEGs is shown, with red indicating upregulated genes and blue indicating downregulated genes. (**B**) GO and KEGG pathway enrichment analyses of the DEGs are included. (**C**-**H**) Gene set enrichment analysis (GSEA) functional enrichment analyses are presented.

Using the median expression as a cut-off, we identified a total of 21,539 DEGs, which included 15,906 upregulated genes and 5,633 downregulated genes (Fig. 4A). By utilising DESeq2 (v1.26.0) in conjunction with the TCGA data, we identified 1,753 significant genes (with logFC > 1.0 or < −1.0 and *P* < 0.05) associated with HROB expression when the low (0–50%) and high (50–100%) expression groups were compared. Subsequently, the GO enrichment analysis highlighted several biological processes (BP), which notably included organelle fission, nuclear division, and chromosome segregation. In terms of cellular components (CC), we observed significant enrichment in chromosomal regions, condensed chromosomes, and centromeric regions. The molecular functions (MF) were mainly associated with hormone activity, cytoskeletal motor activity, and microtubule motor activity. KEGG pathway analysis^18,19^ revealed significant enrichment in genes related to neuroactive ligand-receptor interactions, the cell cycle, and nicotine addiction, thereby indicating a potential role for HROB in regulating the cell cycle (Fig. 4B). To more thoroughly elucidate the biological roles of HROB, GSEA revealed that higher HROB expression was linked to various aspects of the cell cycle, including the resolution of sister chromatid cohesion (Fig. 4C); mitotic G2/G2M phases (Fig. 4D); cell cycle checkpoints (Fig. 4E); the mitotic spindle checkpoint (Fig. 4F); and the functions of the retinoblastoma gene in cancer (Fig. 4G) and the cell cycle (Fig. 4H). These findings indicate that HROB could play a crucial role in regulating the cell cycle and checkpoint control in LUAD.

### The relationship between HROB expression and immune cell infiltration in LUAD

**Fig. 5:**
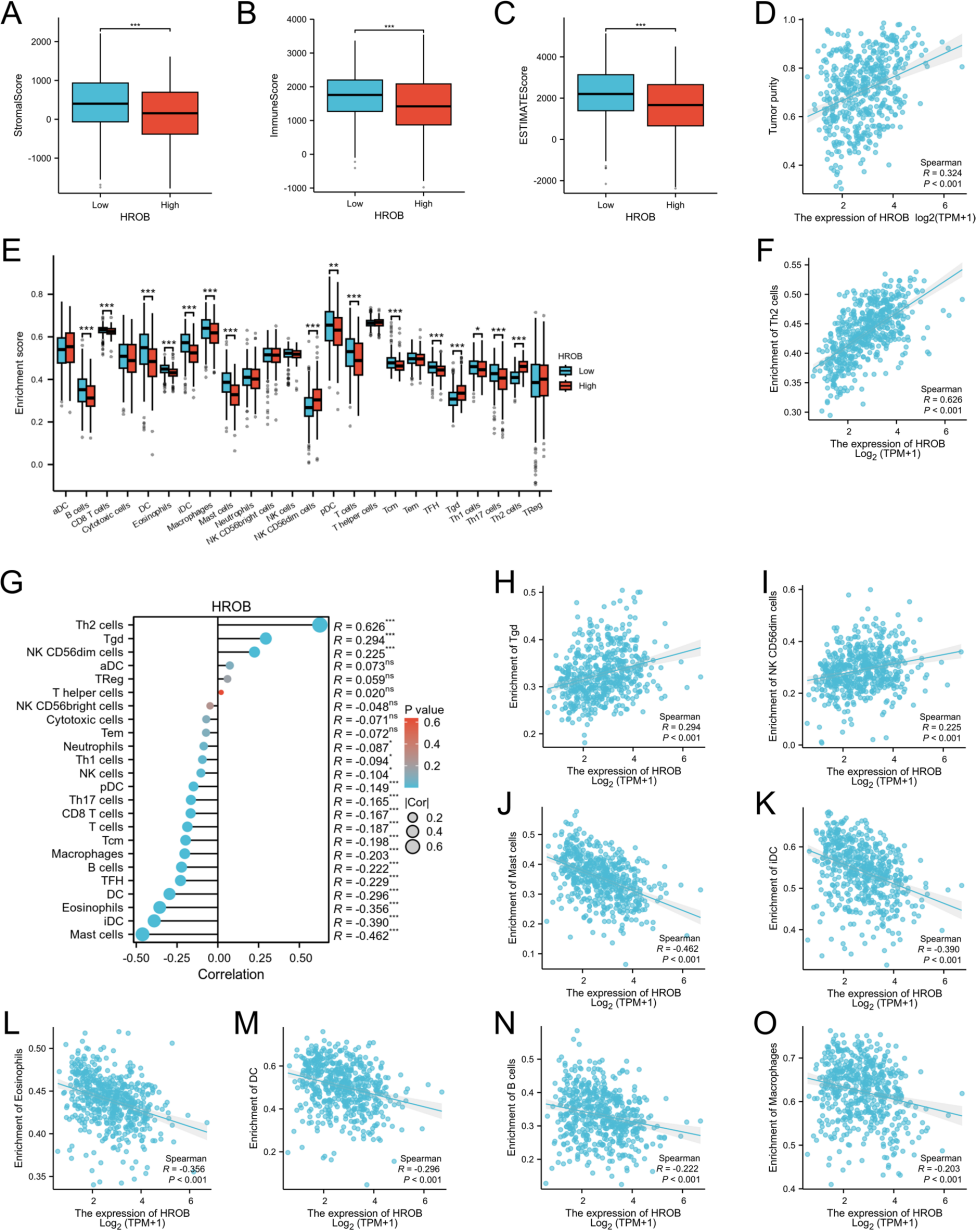
The relationships between HROB expression and immune cell infiltration were explored in LUAD. (**A**-**C**) Comparisons of the StromalScore, ImmuneScore, and EstimateScore between the high- and low-HROB expression groups are provided. (**D**) Correlations between HROB expression and tumour purity are provided. (**E**) Immune cell enrichment scores in the high- versus low-HROB expression groups were compared. (**G**) A correlation analysis between the relative abundances of 24 immune cells and HROB expression is shown, with dot sizes representing the absolute value of Spearman R. (**F**, **H**-**O**) Scatterplots are illustrated: (1) positive correlations between HROB expression and Th2, Tgd, and NK CD56dim cells; (2) negative correlations with mast cells, immature dendritic cells (iDCs), eosinophils, dendritic cells (DCs), B cells, and macrophages. Statistical significance is denoted as follows: ∗*P* < 0.05; ∗∗*P* < 0.01; and ∗∗∗*P* < 0.001.

Our analysis revealed a significant reduction in stromal scores (as illustrated in Fig. 5A), along with decreases in immune scores (Fig. 5B) and estimated scores (Fig. 5C), for LUAD patients with high HROB expression. Correlation analysis revealed a positive correlation between HROB expression and tumour purity (*R* = 0.324, *P* < 0.001) (Fig. 5D). These findings indicate a lower presence of stromal cells in the tumour microenvironment, which is correlated with a poorer prognosis. Additionally, we observed that Th2, Tgd, and CD56dim cells were significantly more abundant in the high HROB expression group. In contrast, the low HROB expression group demonstrated more mast cells, immature dendritic cells (iDCs), eosinophils, dendritic cells (DCs), TFH cells, B cells, and macrophages, as shown in Fig. 5E and G. Furthermore, the results revealed a strong positive correlation between HROB expression and the infiltration of Th2 cells (*R* = 0.626) (Fig. 5F), whereas significant negative correlations were observed between HROB expression and the infiltration of mast cells (*R* = −0.462) (Fig. 5J) and other immune cell types, as depicted in Fig. 5K and O. These findings suggest that HROB may play a role in modulating immune infiltration in LUAD by affecting the distribution of different immune cell subtypes.

### Coexpression analysis of genes with HROB and the PPI network in LUAD

**Fig. 6:**
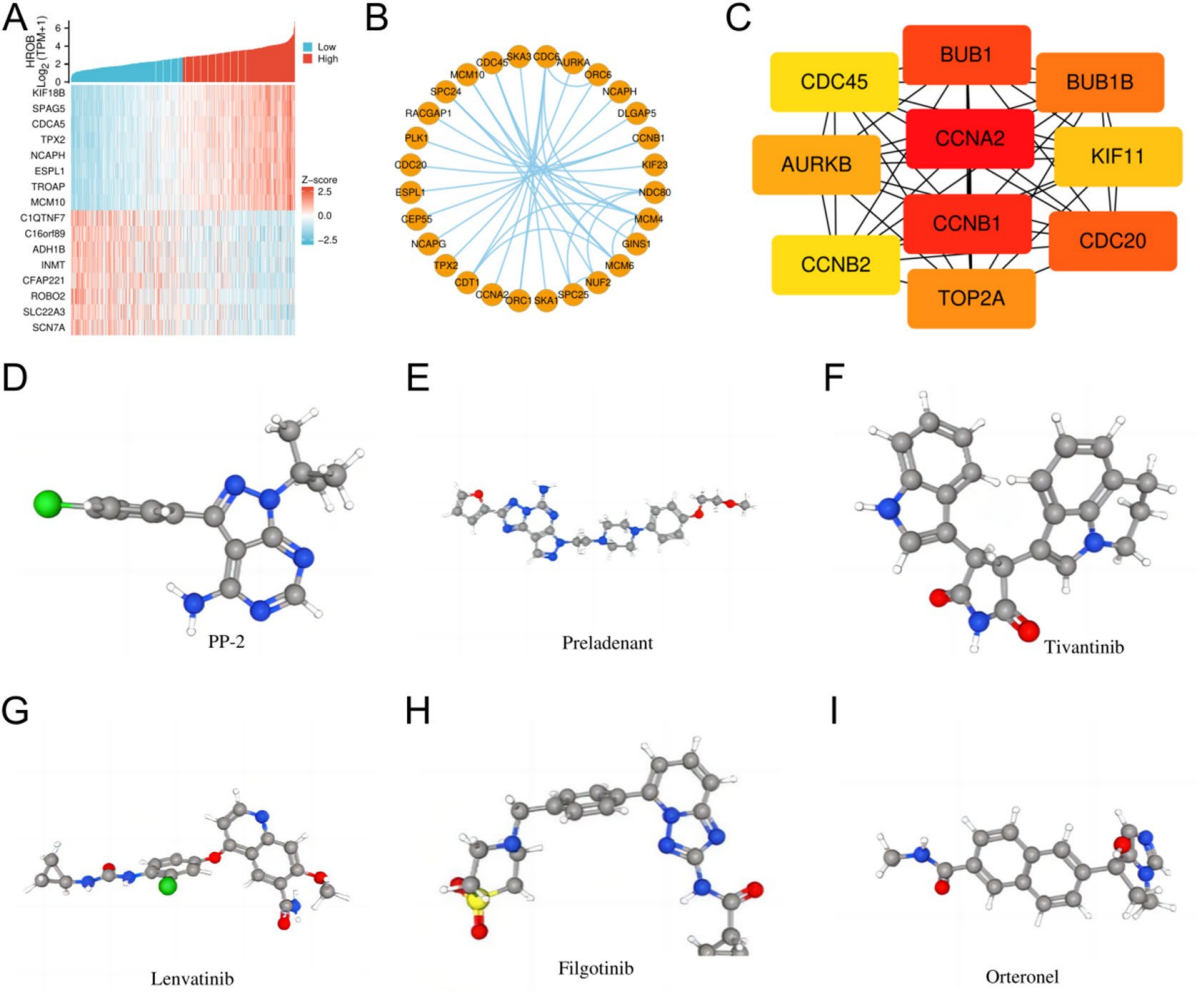
The genes coexpressed with HROB were assessed, protein-protein interactions (PPIs) were analysed, hub genes in lung adenocarcinoma (LUAD) were identified, and small molecular therapeutic agents were determined. (**A**) Sixteen genes related to HROB in LUAD (categorised into 8 positively correlated genes and 8 negatively correlated genes) are displayed in the heatmap. (**B**) The top 30 protein-coding genes that exhibit both positive and negative associations with HROB are illustrated by the PPI network. (**C**) The top 10 hub genes within the PPI network are shown. (**D**-**I**) Chemical structures of six potential therapeutic small molecules are illustrated, including (**D**) PP-2, (**E**) preladenant, (**F**) tivantinib, (**G**) lenvatinib, (**H**) filgotinib, and (**I**) orteronel.

The coexpression heatmap revealed the 16 genes that are most positively and negatively associated with HROB (Fig. 6A). PPI network analysis was performed on the top 108 protein-coding genes correlated with HROB, and the following ten hub genes were identified: CCNA2, CCNB1, BUB1, CDC20, BUB1B, TOP2A, AURKB, KIF11, CDC45, and CCNB2 (Fig. 6B and C). These hub genes are involved in the cell cycle and mutations associated with tumours, with increased expression levels being observed in LUAD (Supplementary Information: Fig. S1). Kaplan-Meier survival analysis revealed that the high expression of these ten hub genes is linked to poorer overall survival outcomes (Supplementary Information: Fig. S2). Additionally, ROC analysis confirmed the diagnostic potential of these hub genes in LUAD (Supplementary Information: Fig. S3). By integrating these hub genes with HROB, the precision of prognostic predictions for LUAD patients may be improved, thereby enriching the molecular profile of the disease.

### Identification of potential therapeutic small-molecule agents based on DEGs

We identified and selected the six small molecules with the highest absolute enrichment values, which are listed in Table 4. These molecules include PP-2, preladenant, tivantinib, lenvatinib, filgotinib, and orteronel. We collected detailed information about these compounds, including their structures. The three-dimensional conformations of these six small molecules are illustrated in Fig. 6D and I. Each of these agents may provide potential therapeutic benefits for the treatment of LUAD.

**Table 4:** Six potential therapeutic molecules identified via shared DEGs.

Characteristics	Total (*N*)	Univariate analysis		Multivariate analysis	
		Hazard ratio (95% CI)	*P* value	Hazard ratio (95% CI)	*P* value
Pathological T stage	527				
T1	176	Reference		Reference	
T2	285	1.507 (1.059–2.146)	**0.023**	0.988 (0.590–1.656)	0.964
T3	47	2.964 (1.762–4.986)	**< 0.001**	1.924 (0.884–4.187)	0.099
T4	19	3.357 (1.767–6.376)	**< 0.001**	1.425 (0.574–3.537)	0.445
Pathological N stage	514				
N0	345	Reference		Reference	
N1	96	2.293 (1.632–3.221)	**< 0.001**	1.560 (0.987–2.468)	0.057
N2&N3	73	2.993 (2.057–4.354)	**< 0.001**	2.517 (1.496–4.235)	**< 0.001**
Pathological M stage	381				
M0	356	Reference		Reference	
M1	25	2.176 (1.272–3.722)	**0.005**	1.649 (0.781–3.483)	0.189
Primary therapy outcome	442				
CR	328	Reference		Reference	
PR	5	2.636 (0.646–10.759)	0.177	7.967 (1.850–34.308)	**0.005**
SD	38	1.087 (0.546–2.163)	0.813	1.177 (0.529–2.617)	0.690
PD	71	3.755 (2.615–5.391)	**< 0.001**	4.419 (2.793–6.992)	**< 0.001**
Smoker	516				
No	74	Reference			
Yes	442	0.942 (0.625–1.420)	0.775		
Gender	530				
Female	283	Reference			
Male	247	1.087 (0.816–1.448)	0.569		
Age	520				
≤ 65	257	Reference			
> 65	263	1.216 (0.910–1.625)	0.186		
HROB	530				
Low	267	Reference		Reference	
High	263	1.616 (1.208–2.163)	**0.001**	1.815 (1.205–2.734)	**0.004**

### HROB expression in A549 cells and validation of gene Silencing efficacy

**Fig. 7:**
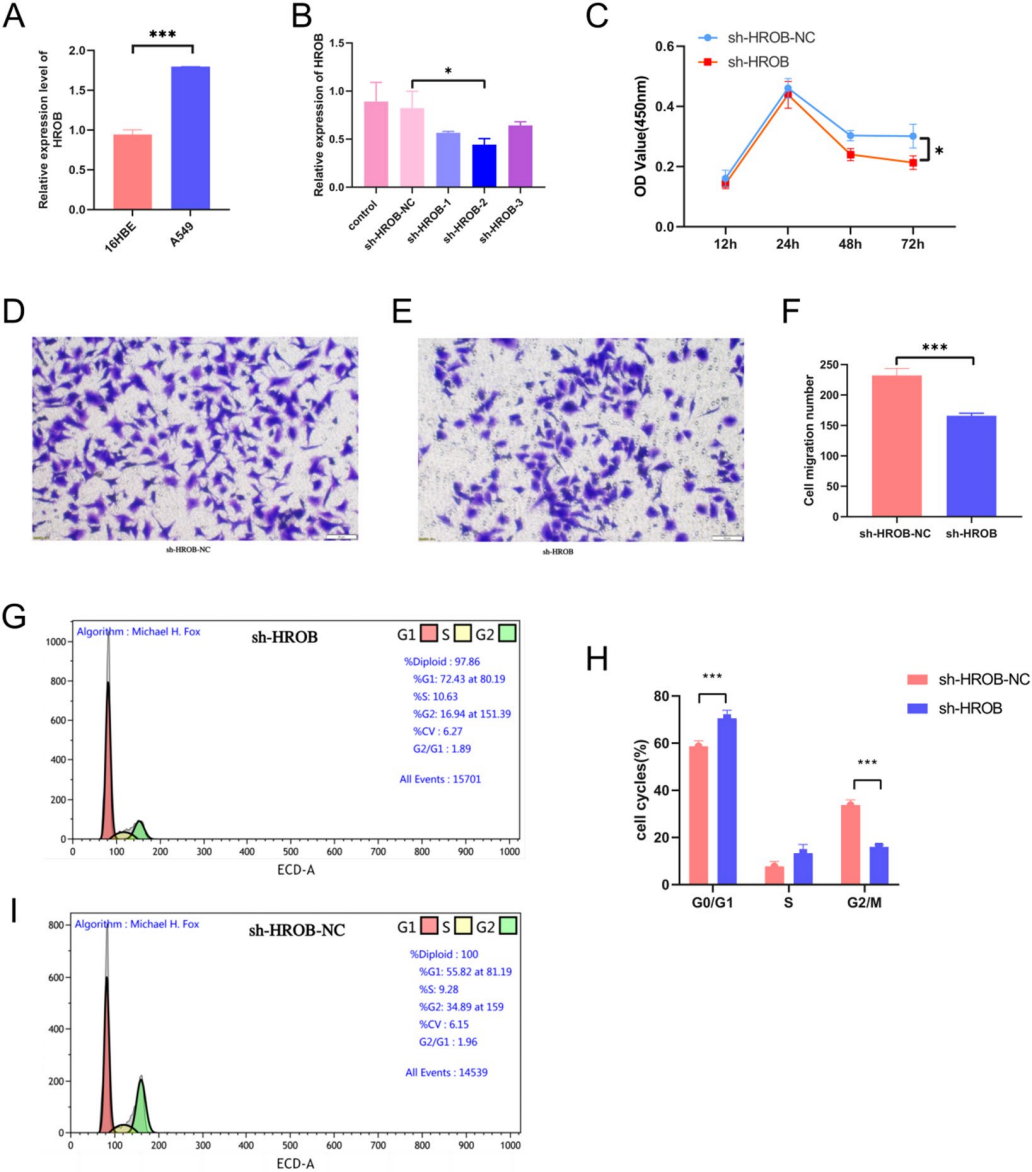
The functional characteristics of HROB were analysed in lung cancer cells. (**A**) The levels of HROB expression were evaluated in both normal cells and A549 cells. (**B**) The expression levels of HROB in A549 cells were analysed after the application of short hairpin RNAs (shRNAs) targeting HROB. (**C**) A proliferation assay for A549 cells was performed by employing the CCK-8 method. (**D**-**F**) Findings from the Transwell assay revealed the extent of invasion of lung adenocarcinoma cells after HROB silencing. (**G**-**I**) Flow cytometry analysis revealed that a reduction in HROB expression affected the progression of the cell cycle in A549 cells. Statistical significance is denoted as follows: ∗*P* < 0.05; ∗∗*P* < 0.01; and ∗∗∗*P* < 0.001 compared with the control group.

The expression levels of HROB in both the A549 and 16HBE cell lines were significantly greater in A549 cells (*P* < 0.05), as demonstrated in Fig. 7A. To specifically decrease HROB expression in A549 cells, we designed and evaluated three different short hairpin RNAs (shRNAs): sh-HROB-1, sh-HROB-2, and sh-HROB-3. PCR analysis revealed that compared with the other shRNAs, sh-HROB-2 was the most effective at reducing HROB expression, as shown in Fig. 7B.

### The impact of HROB gene Silencing on A549 cell proliferation, invasion, and migration

The results of the CCK-8 assay indicated that shRNA significantly inhibited cell growth. Specifically, at the 72-hour time point, compared with control cells, A549 cells transfected with sh-HROB-2 exhibited a significantly reduced proliferative capacity (Fig. 7C, *P* < 0.05). Additionally, the silencing of HROB via sh-HROB-2 led to a notable decrease in the invasion of A549 cells compared with that in the sh-NC control group (*P* < 0.001; Fig. 7D and F). These results suggest that HROB plays a role in enhancing the invasive characteristics of LUAD.

### Cell cycle analysis

The findings of the cell cycle analysis revealed that a greater percentage of A549 cells transfected with sh-HROB-2 were in the G1 phase compared to those in the sh-NC control group, with values of 72.43% versus 55.82% being observed, respectively. Conversely, the proportion of cells in the G2 phase was significantly lower in the transfected group (16.94%) than in the control group (34.89%). No significant differences were noted in the S phase (Fig. 7G and I). These results indicate that HROB is essential for facilitating cell cycle progression.

## Discussion

Despite significant advancements in diagnostic and therapeutic strategies for LUAD over the past two decades, prevalent challenges remain for patients with this disease^20^.

This study focused on the overexpression of HROB in LUAD and its association with poor patient outcomes. Previous studies have demonstrated the important role of HROB in DNA damage repair mechanisms^12^. HROB is categorised as an OB-fold-containing factor that is involved in homologous recombination, and it aids in the recruitment of the MCM8-MCM9 helicase to sites of DNA damage, which correspondingly facilitates DNA synthesis^11^. Furthermore, HROB may affect tumour progression through pathways related to cell proliferation^13^. Our results revealed that HROB expression is markedly increased in LUAD and is correlated with unfavourable prognostic outcomes. Similar findings have been observed in other cancers^13,15^. These findings suggest that HROB may play a critical role in cancer development across different tumour types.

Our additional findings revealed that the increased expression of HROB is associated with more advanced disease stages and poorer survival outcomes across various analyses, including OS, DSS, and PFI. Logistic regression analysis further confirmed that HROB serves as an independent prognostic marker for OS. Additionally, the results of the ROC curve analysis indicated a larger AUC, which reflects a high level of predictive accuracy for HROB expression in LUAD. Compared with traditional clinicopathological parameters, a nomogram that included HROB expression demonstrated enhanced prognostic prediction. Together, these findings highlight the potential of HROB as an independent prognostic biomarker in LUAD, which is consistent with the findings of other studies^15,21^.

The exploration of HROB alongside established prognostic factors (such as smoking history) provides valuable insights. A strong correlation between high HROB expression and poor outcome was detected in smokers with LUAD, as evidenced by significantly lower survival rates. Furthermore, the total number of pack-years smoked was associated with HROB expression. This highlights the potential of HROB as an additional biomarker for risk assessment in patients, especially in relation to smoking, which is a well-known risk factor for lung cancer^22^. Moreover, the relationships of HROB expression with clinical characteristics such as pathological T stage, pathological stage, age, primary therapy outcomes, OS events, and DSS events underscore the potential usefulness of HROB in categorising patients based on risk profiles. These findings suggest that higher levels of HROB expression are associated with worse clinical outcomes, thereby aiding clinical decision-making, with these results being similar to findings in other tumour types^13,15^. These findings suggest that HROB could serve not only as a prognostic biomarker but also as a promising therapeutic target.

We conducted a thorough differential expression analysis to identify genes that are linked to HROB expression in LUAD. GO enrichment analysis revealed key biological processes, such as organelle fission and chromosome segregation, which are crucial for understanding how HROB influences the growth potential of LUAD cells. Notably, these DEGs significantly affect cell cycle regulation. Additionally, KEGG pathway analysis revealed that the DEGs associated with HROB are involved in nuclear division and cell cycle processes^23^. Furthermore, GSEA revealed a link between increased HROB expression and various biological processes, including the resolution of sister chromatid cohesion, the mitotic process, cell cycle checkpoints, and the mitotic spindle checkpoint. These insights provide a foundation for further research into the biological functions of HROB and its associated genes, which could lead to the identification of new therapeutic targets for LUAD treatment. To explore coexpressed genes related to HROB and their functional networks, we performed a comprehensive PPI network analysis. This analysis emphasised ten key hub genes (CCNA2, CCNB1, BUB1, CDC20, BUB1B, TOP2A, AURKB, KIF11, CDC45, and CCNB2), which are closely linked to the survival of LUAD patients. Furthermore, recent studies have demonstrated that alterations in cell cycle genes may be involved in the development of osimertinib resistance^24^. Overall, these hub genes and HROB are vital components of the cell cycle and may represent innovative therapeutic targets for LUAD^25–34^.

The results of the bioinformatics analysis were validated by our experimental results. Cell cycle assays revealed notable effects on the cell cycle, thereby further clarifying the manner by which HROB influences the proliferation, migration, and invasion of LUAD cells. Proper regulation of the cell cycle is crucial for maintaining normal cellular functions and tissue homeostasis. Moreover, disruptions in this regulation are closely associated with the development and progression of various diseases, especially cancers^35^. Tumour cells often exhibit a shorter duration in the G1 phase and a higher percentage of cells in the S and G2/M phases, which accelerates their division and proliferation^36,37^. Our findings indicated that the silencing of HROB led to an increased population of cells in the G1 phase and a decreased percentage in the G2 phase, thus supporting this notion. However, the flow cytometry results revealed no significant difference in the S phase in our experiments, which may be attributed to the varying effects of genes on the cell cycle in different cell types, with differences in genetic backgrounds, signalling pathways, and the utilised detection methods being noted. Furthermore, the influence of HROB may be particularly significant during the initial phases of the cell cycle, rather than in the course of DNA replication. The overactivation of the CDK4/6-cyclin D complex propels tumor cells to prematurely enter the S phase from the G1 phase, a phenomenon frequently observed as a hallmark of cell cycle dysregulation in tumors^38^. It is postulated that HROB may expedite tumor proliferation by modulating cyclins or engaging in the oversight of the G2/M checkpoint. The interaction and functionality with associated cyclin complexes warrant further exploration through co-immunoprecipitation and targeted interference methodologies. These findings position HROB as a crucial player in DNA damage repair mechanisms and the regulation of cell cycle dynamics^21,39^. Based on this role, HROB likely promotes tumour therapy resistance in LUAD by orchestrating the collaborative regulation of cell cycle genes and DNA repair molecules, particularly in the context of aberrant EGFR signalling axes. Thus, the elucidation of the mechanistic interplay between HROB and EGFR-driven pathways (specifically in terms of cell cycle control, the DNA damage response, and drug resistance) may provide novel theoretical foundations for LUAD molecular subtyping, prognostic stratification, and personalised therapeutic targeting^12,24^.

The tumour microenvironment plays a crucial role in tumour progression and affects patient outcomes^40^. Recent studies have elucidated the complex immune landscape of LUAD, highlighting the involvement of various immune cell types that can either promote or inhibit tumour growth^41,42^. An understanding of these interactions is essential for developing targeted immunotherapies^43–45^. In our research, we investigated HROB expression levels in LUAD and their relationship with immune cell infiltration. We observed that higher HROB expression can reduce the immune and stromal score of patients and increase the tumor purity, thus suggesting that HROB plays a key role in shaping the immune microenvironment in LUAD. Notably, HROB expression is strongly positively correlated with Th2 cell infiltration, thereby indicating that HROB may promote a Th2-skewed immune response. This response is characterised by an abundance of Th2 cells that secrete cytokines such as IL-4 and IL-13, which are known to enhance the development of a tumour-promoting M2 or M2-like phenotype in tumour-associated macrophages^46^. Conversely, we observed a negative correlation between HROB expression and mast cell infiltration. These findings suggest that HROB may be linked to reduced mast cell activity, which typically includes stimulating tumour cell proliferation; creating an immunosuppressive tumour microenvironment; promoting angiogenesis and lymphangiogenesis; and facilitating invasion and metastasis^47^. Additionally, the expression level of HROB exhibits a notable negative correlation with the infiltration of dendritic cells (DCs), which are pivotal in orchestrating T cell-mediated anti-tumor immunity^48^. In summation, HROB emerges as a pivotal factor in establishing an immunosuppressive microenvironment through the modulation of DNA damage response signaling pathways^21,39^. Furthermore, it may facilitate tumor immune evasion by intricately altering cytokine secretion patterns or disrupting the recruitment of immune cells. HROB stands as a crucial nexus, intertwining the aberrant cell cycle with the reprogramming of the immune microenvironment, a process integral to the progression of LUAD, thereby offering a promising molecular foundation for targeted and combinatorial immunotherapeutic strategies.

Finally, our investigation into potential therapeutic small molecules based on DEGs resulted in the identification of several candidates with significant clinical implications for treating LUAD. We identified six small molecules, including PP-2, preladenant, tivantinib, lenvatinib, filgotinib, and orteronel, which demonstrated high enrichment values. The exploration of their mechanisms could provide essential insights into their therapeutic efficacy in LUAD, especially with respect to molecular docking simulation results (such as AutoDock Vina scores). Although some clinical trials validating the application of these agents have established their integration into LUAD standard treatment protocols^49^, the efficacy and safety of these agents must be validated through preclinical animal studies and subsequently confirmed by large-scale clinical trials.

This study has several limitations. One limitation involves the lack of clinical validation and the relatively small sample size, which could restrict the applicability of the results to larger populations. Additionally, the reliance on publicly available datasets may introduce bias because of batch effects and differences in how the data were collected. The absence of comprehensive in vitro and in vivo experiments to support bioinformatics analyses represents another limitation. Additionally, no specific evaluation has been performed regarding HROB heterogeneity across distinct LUAD molecular subtypes, such as the KRAS/STK11 comutation subgroup, which may undermine the prognostic relevance of these subtypes. For the predicted small-molecule candidates, molecular docking simulations with the HROB protein and the corresponding binding-energy calculations were not performed. Therefore, three approaches to propel future research are planned: firstly, it is imperative to validate the correlation between HROB expression and survival outcomes through immunohistochemical and transcriptomic assays in LUAD specimens from diverse populations and different clinical stages across multiple centers, thereby elucidating its independence and complementarity with extant prognostic factors. Secondly, an exploration into the ramifications of HROB knockdown or inhibition on tumor proliferation, metastasis, and drug sensitivity in animal models is warranted, with its interaction mechanisms with pathways such as EGFR. Lastly, studies encompassing molecular binding, pharmacodynamics, pharmacokinetics, and safety assessments of candidate small molecules, including tivantinib^50^.

In summary, our study highlights a significant association between the overexpression of HROB and poor outcomes in lung adenocarcinoma, thereby establishing HROB as an important independent prognostic biomarker. The complex interplay between HROB expression, cancer traits, changes in the immune microenvironment, and possible therapeutic targets indicates that HROB could serve as a vital biomarker to guide treatment approaches. More research is needed to explore the role of HROB in LUAD in the future.

## Supplementary Information

Below is the link to the electronic supplementary material.

Supplementary Material 1

Supplementary Material 2

## Data Availability

The datasets are available in the TCGA dataset (https://tcga-data.nci.nih.gov/TCGA/) and GEO dataset (https://www.ncbi.nlm.nih.gov/geo/query/acc.cgi?acc=). The data that were analysed during the current study are available from the Xiantao academic tool (xiantaozi.com/products/apply/c0b6febb-52dd-4525-970a-61bbe9e263ff/collect). All of the experiments were performed in accordance with the relevant regulations and followed the manufacturers’ instructions. Data are available on reasonable request. All data relevant to the study are included in the article or uploaded as supplemental information.
