# Supplementary material for: A comprehensive study integrating bioinformatics analysis and experimental results on HROB as a potential biomarker for the prognosis of lung adenocarcinoma
Source: Sci Rep. 2026 Jan 12;16:5056. doi: 10.1038/s41598-026-35798-7 (PMC12876862; doi:10.1038/s41598-026-35798-7)
Supplement: Supplementary file 1 — Supplementary Material 1 [file 41598_2026_35798_MOESM1_ESM.pdf]

A Comprehensive Study Integrating Bioinformatics Analysis and Experimental Results on HROB as a Potential Biomarker for the Prognosis of Lung Adenocarcinoma

Fayan Zhang[1], Xiao Liu[2], Shengyu Zhou[3,2]*

1 College of Medicine, Shandong University of Traditional Chinese Medicine, Jinan, China

2 Department of Respiratory and Critical Care Medicine, Qilu Hospital, Cheeloo College of Medicine, Shandong University, Jinan, China

3 Clinical Nursing Department, School of Nursing and Rehabilitation, Cheeloo College of Medicine, Shandong University, Jinan, China; Department of Respiratory and Critical Care Medicine, Qilu Hospital, Cheeloo College of Medicine, Shandong University, Jinan, China

***Correspondence:**

Corresponding Author: Shengyu Zhou

shengyugood@163.com

Table S1. Differential HROB expression across various tumor tissues

Tumor	GroupI	GroupJ	Statistic	Difference (J–I)	95% CI	P value
BLCA	Normal	Tumor	616	1.8069	1.3754 – 2.1722	5.24e-10
BRCA	Normal	Tumor	3.222e+04	0.53027	0.40982 – 0.65076	1.23e-17
CESC	Normal	Tumor	0	2.853	2.1602 – 3.5372	0.0029
CHOL	Normal	Tumor	0	2.2891	1.7351 – 2.7162	2.82e-09
COAD	Normal	Tumor	804	1.3944	1.2281 – 1.5636	1.58e-22
ESCA	Normal	Tumor	56	2.2359	1.6618 – 2.7752	2.05e-07
GBM	Normal	Tumor	7	1.6641	1.2007 – 2.0759	0.0002
HNSC	Normal	Tumor	2215	1.3832	1.1181 – 1.6434	1.27e-18
KICH	Normal	Tumor	1172	-0.2963	-0.43249 – -0.14193	0.0012
KIRC	Normal	Tumor	3471	0.84323	0.74213 – 0.94029	8.64e-30
KIRP	Normal	Tumor	1086	0.75249	0.58478 – 0.94076	1.09e-12
LIHC	Normal	Tumor	1199	1.1318	0.92994 – 1.3589	1.31e-23
LUAD	Normal	Tumor	1033	1.6472	1.4071 – 1.8719	3.91e-32
LUSC	Normal	Tumor	124	2.7667	2.5616 – 2.9472	2.49e-30
PAAD	Normal	Tumor	212	0.30719	-0.17589 – 0.78845	0.1649
PCPG	Normal	Tumor	59	0.45282	0.099594 – 0.97089	0.0199
PRAD	Normal	Tumor	1.466e+04	-0.093104	-0.21733 – 0.027401	0.1362
READ	Normal	Tumor	106	1.2161	0.82733 – 1.5905	3.68e-06
STAD	Normal	Tumor	775	1.5992	1.3243 – 1.8676	2.86e-16
THCA	Normal	Tumor	6527	0.4124	0.31343 – 0.50228	8.85e-13
UCEC	Normal	Tumor	1155	1.7203	1.4663 – 1.9627	2.21e-18

Legend

Table S1. Differential HROB expression between normal and tumor tissues across 21 cancer types was assessed using the Wilcoxon rank-sum test. The Statistic column represents the test statistic value. The Difference (log2FC) column is defined as follows: negative values indicate low expression in tumor tissue compare to normal tissue, while positive values indicate high expression.

Abbreviations:

CI: confidence interval; BLCA: Bladder Urothelial Carcinoma; BRCA: Breast invasive Carcinoma; CESC: Cervical squamous cell carcinoma and endocervical adenocarcinoma; CHOL: Cholangiocarcinoma; COAD: Colon adenocarcinoma; ESCA: Esophageal carcinoma; GBM: Glioblastoma multiforme; HNSC: Head and Neck squamous cell carcinoma; KICH: Kidney Chromophobe; KIRC: Kidney renal clear cell carcinoma, KIRP: Kidney renal papillary cell carcinoma; LIHC: Liver hepatocellular carcinoma; LUAD: Lung adenocarcinoma; LUSC: Lung squamous cell carcinoma; PAAD: Pancreatic adenocarcinoma; PCPG: Pheochromocytoma and Paraganglioma; PRAD: Prostate adenocarcinoma; READ: Rectum adenocarcinoma; STAD: Stomach adenocarcinoma; THCA: Thyroid carcinoma; UCEC: Uterine Corpus Endometrial Carcinoma

Fig. S1

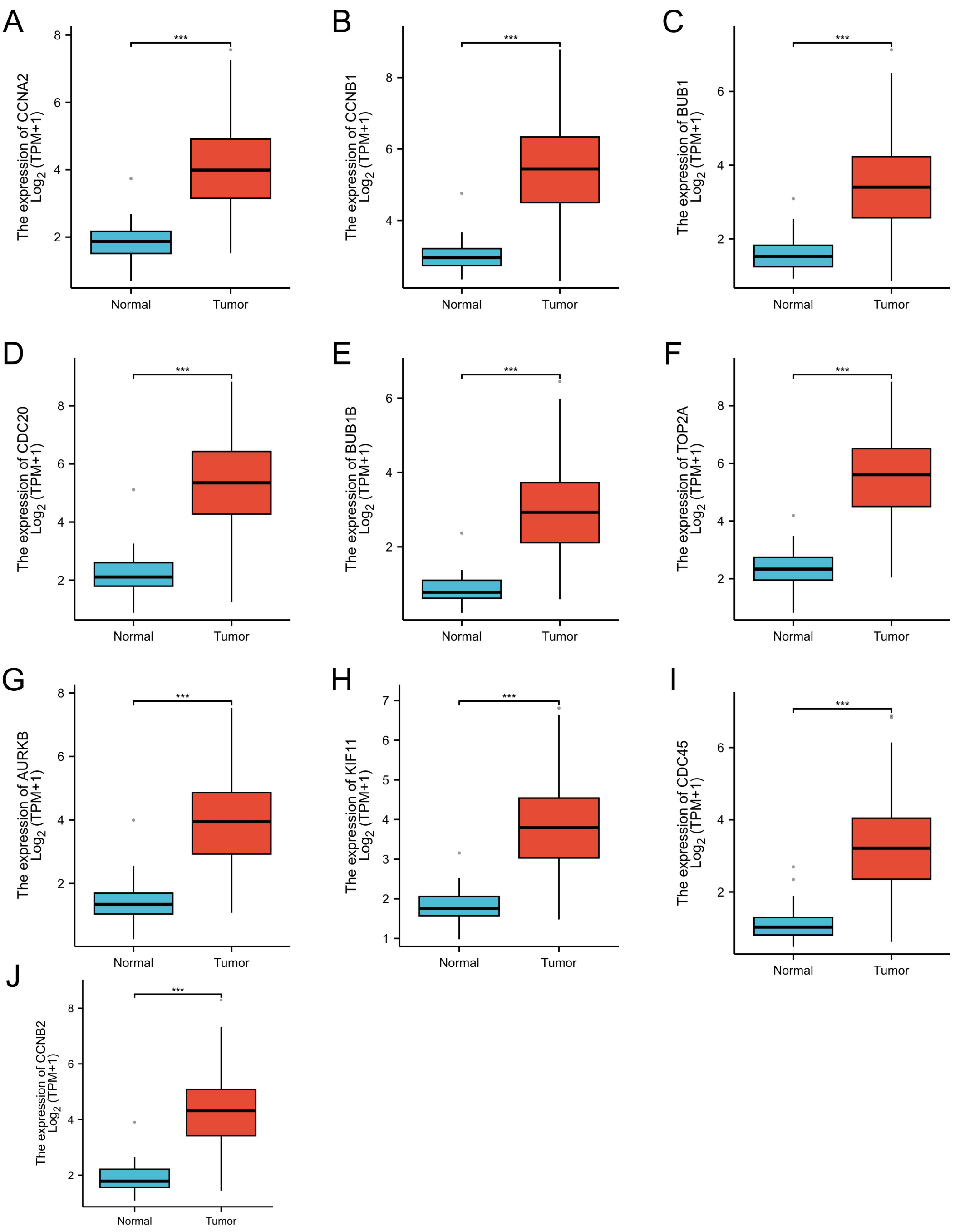

Fig. S1. Comparison of expression levels of 10 hub genes between normal and tumor tissues. This figure consists of 10 subpanels (A-J), each corresponding to one of the 10 hub genes (CCNA2, CCNB1, BUB1, CDC20, BUB1B, TOP2A, AURKB, KIF11, CDC45, CCNB2). The horizontal axis represents tissue types; the vertical axis represents gene expression levels. All 10 hub genes show higher expression levels in tumor tissues than in normal tissues, indicating significant upregulation of these genes in tumor tissues. Statistical significance is indicated as follows: ＊ $p < 0.05$; ＊＊ $p < 0.01$; and ＊＊＊ $p < 0.001$ compared with the control group.

Fig. S2

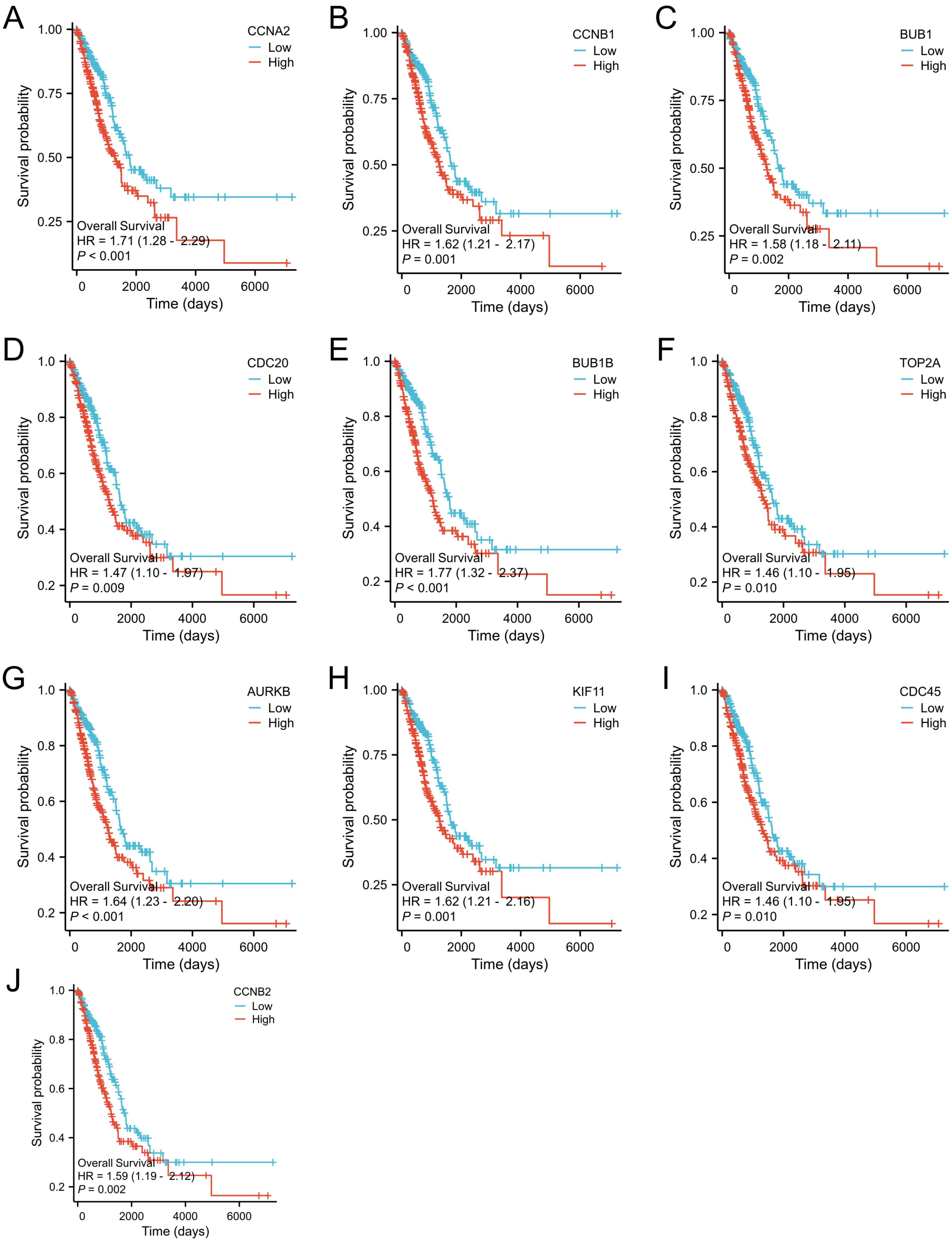

Fig. S2. Kaplan - Meier survival curves for overall survival in relation to the expression levels of different genes. (A) CCNA2; (B) CCNB1; (C) BUB1; (D) CDC20; (E) BUB1B; (F) TOP2A; (G) AURKB; (H) KIF11; (I) CDC45; (J) CCNB2. Blue curves represent the low - expression groups, and red curves represent the high - expression groups. HR (hazard ratio) and P values for overall survival differences between the high - and low - expression groups are shown in each panel.

Fig. S3

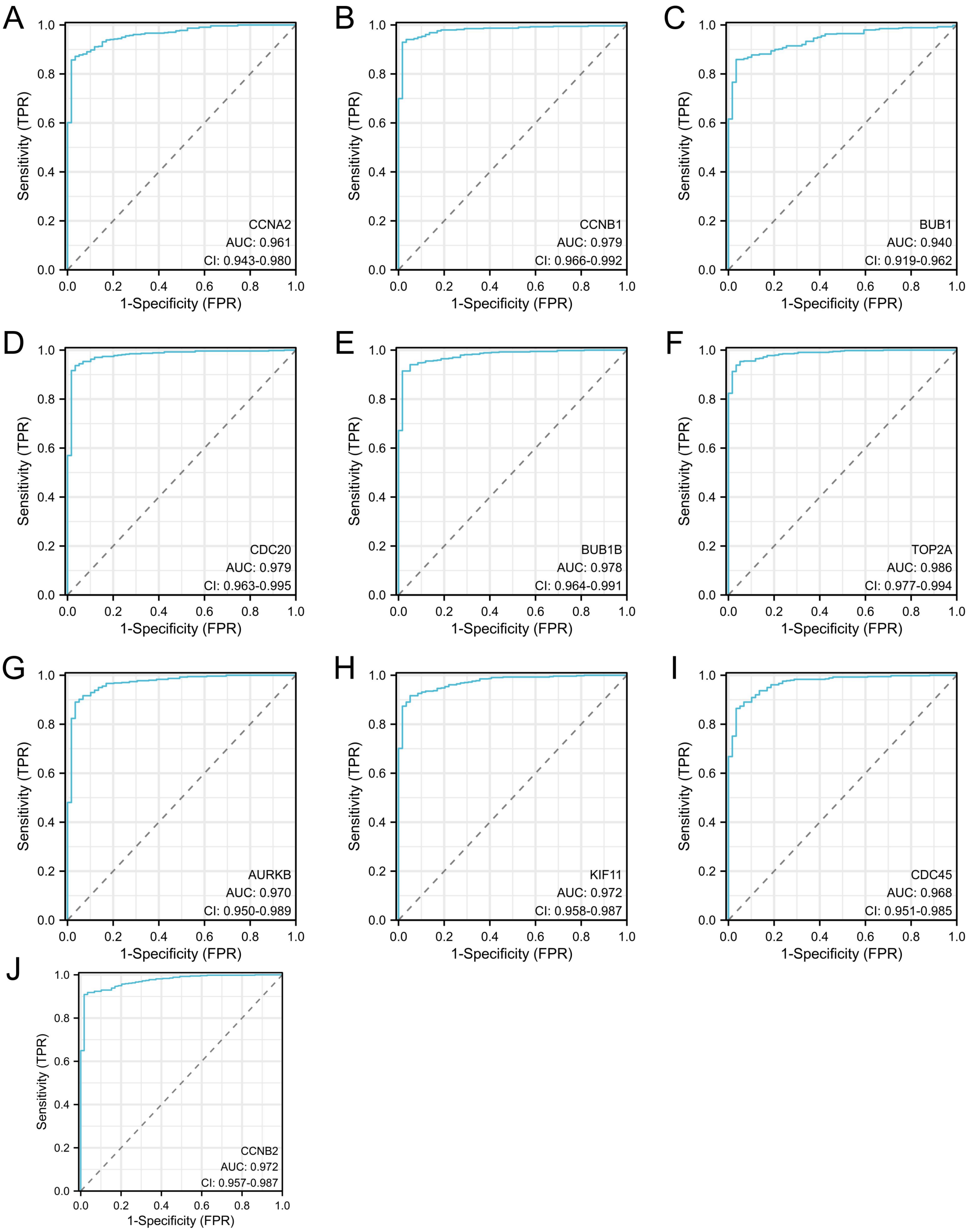

Fig. S3. Receiver Operating Characteristic (ROC) curves of 10 hub genes

This figure consists of 10 subpanels (A-J), each corresponding to a specific hub gene and presenting its ROC curve along with key diagnostic performance indicators. (A) CCNA2; (B) CCNB1; (C) BUB1; (D) CDC20; (E) BUB1B; (F) TOP2A; (G) AURKB; (H) KIF11; (I) CDC45; (J) CCNB2. The vertical axis represents Sensitivity (True Positive Rate, TPR), and the horizontal axis represents 1-Specificity (False Positive Rate, FPR). All 10 hub genes show extremely high diagnostic value, with AUC values ranging from 0.940 to 0.986.
