# Supplementary material for: A comprehensive study integrating bioinformatics analysis and experimental results on HROB as a potential biomarker for the prognosis of lung adenocarcinoma
Source: Sci Rep. 2026 Jan 12;16:5056. doi: 10.1038/s41598-026-35798-7 (PMC12876862; doi:10.1038/s41598-026-35798-7)
Supplement: Supplementary file 2 — Supplementary Material 2 [file 41598_2026_35798_MOESM2_ESM.docx]

# **Table S1.** Differential HROB expression across various tumor tissues

| Tumor | GroupI | GroupJ | Statistic | Difference (J–I) | 95% CI | P value |
| --- | --- | --- | --- | --- | --- | --- |
| BLCA | Normal | Tumor | 616 | 1.8069 | 1.3754 – 2.1722 | 5.24e-10 |
| BRCA | Normal | Tumor | 3.222e+04 | 0.53027 | 0.40982 – 0.65076 | 1.23e-17 |
| CESC | Normal | Tumor | 0 | 2.853 | 2.1602 – 3.5372 | 0.0029 |
| CHOL | Normal | Tumor | 0 | 2.2891 | 1.7351 – 2.7162 | 2.82e-09 |
| COAD | Normal | Tumor | 804 | 1.3944 | 1.2281 – 1.5636 | 1.58e-22 |
| ESCA | Normal | Tumor | 56 | 2.2359 | 1.6618 – 2.7752 | 2.05e-07 |
| GBM | Normal | Tumor | 7 | 1.6641 | 1.2007 – 2.0759 | 0.0002 |
| HNSC | Normal | Tumor | 2215 | 1.3832 | 1.1181 – 1.6434 | 1.27e-18 |
| KICH | Normal | Tumor | 1172 | -0.2963 | -0.43249 – -0.14193 | 0.0012 |
| KIRC | Normal | Tumor | 3471 | 0.84323 | 0.74213 – 0.94029 | 8.64e-30 |
| KIRP | Normal | Tumor | 1086 | 0.75249 | 0.58478 – 0.94076 | 1.09e-12 |
| LIHC | Normal | Tumor | 1199 | 1.1318 | 0.92994 – 1.3589 | 1.31e-23 |
| LUAD | Normal | Tumor | 1033 | 1.6472 | 1.4071 – 1.8719 | 3.91e-32 |
| LUSC | Normal | Tumor | 124 | 2.7667 | 2.5616 – 2.9472 | 2.49e-30 |
| PAAD | Normal | Tumor | 212 | 0.30719 | -0.17589 – 0.78845 | 0.1649 |
| PCPG | Normal | Tumor | 59 | 0.45282 | 0.099594 – 0.97089 | 0.0199 |
| PRAD | Normal | Tumor | 1.466e+04 | -0.093104 | -0.21733 – 0.027401 | 0.1362 |
| READ | Normal | Tumor | 106 | 1.2161 | 0.82733 – 1.5905 | 3.68e-06 |
| STAD | Normal | Tumor | 775 | 1.5992 | 1.3243 – 1.8676 | 2.86e-16 |
| THCA | Normal | Tumor | 6527 | 0.4124 | 0.31343 – 0.50228 | 8.85e-13 |
| UCEC | Normal | Tumor | 1155 | 1.7203 | 1.4663 – 1.9627 | 2.21e-18 |

**Legend**

**Table S1.** Differential HROB expression between normal and tumor tissues across 21 cancer types was assessed using the Wilcoxon rank-sum test. The Statistic column represents the test statistic value. The Difference (log2FC) column is defined as follows: negative values indicate low expression in tumor tissue compare to normal tissue, while positive values indicate high expression.

Abbreviations:

CI: confidence interval

BLCA: Bladder Urothelial Carcinoma

BRCA: Breast invasive Carcinoma

CESC: Cervical squamous cell carcinoma and endocervical adenocarcinoma

CHOL: Cholangiocarcinoma

COAD: Colon adenocarcinoma

ESCA: Esophageal carcinoma

GBM: Glioblastoma multiforme

HNSC: Head and Neck squamous cell carcinoma

KICH: Kidney Chromophobe

KIRC: Kidney renal clear cell carcinoma

KIRP: Kidney renal papillary cell carcinoma

LIHC: Liver hepatocellular carcinoma

LUAD: Lung adenocarcinoma

LUSC: Lung squamous cell carcinoma

PAAD: Pancreatic adenocarcinoma

PCPG: Pheochromocytoma and Paraganglioma

PRAD: Prostate adenocarcinoma

READ: Rectum adenocarcinoma

STAD: Stomach adenocarcinoma

THCA: Thyroid carcinoma

UCEC: Uterine Corpus Endometrial Carcinoma
